# Novel Coronavirus Disease 2019 and Subarachnoid Hemorrhage: A Case Report

**DOI:** 10.7759/cureus.7846

**Published:** 2020-04-27

**Authors:** Alexandra Craen, Gideon Logan, Latha Ganti

**Affiliations:** 1 Emergency Medicine, University of Central Florida College of Medicine, Orlando, USA; 2 Emergency Medicine, Envision Physician Services, Nashville, USA; 3 Emergency Medicine, University of Central Florida College of Medicine/Hospital Corporation of America Graduate Medical Education Consortium of Greater Orlando, Orlando, USA; 4 Emergency Medical Services, Polk County Fire Rescue, Bartow, USA

**Keywords:** covid-19, subarachnoid hemorrhage

## Abstract

The novel coronavirus disease 2019 (COVID-19) has had a profound impact on healthcare systems around the world. The emergency department (ED) in particular has become the frontline for the identification and care of these patients. While its effects on respiratory symptoms are well recognized, neurologic manifestations have been rarer. We report the case of a patient who presented in cardiac arrest with the return of spontaneous circulation (ROSC). The patient was found to have subarachnoid hemorrhage and later tested positive for COVID-19.

## Introduction

The novel coronavirus was identified in December of 2019 after an outbreak of pneumonia cases in Wuhan, China. It spread rapidly and was declared a pandemic by the World Health Organization on March 11, 2020 [1]. The disease was designated as COVID-19, caused by the virus termed as severe acute respiratory syndrome coronavirus 2 (SARS-CoV-2) [2]. It has been confirmed in over a million people worldwide at this time. The most common symptoms include fever, cough, and dyspnea, with initial reports from China showing only mild symptoms in 81% of patients. Critical symptoms of the disease, including respiratory failure and shock, were reported in 5% of patients. The overall fatality rate associated with the disease is reported to be 2.3% [3]. Other symptoms reported from around the world include smell and taste abnormalities and gastrointestinal symptoms [4,5]. Neurologic symptoms and intracranial hemorrhages have rarely been reported to date.

In this report, we present the case of a patient who was brought in by paramedics as a case of cardiac arrest with the return of spontaneous circulation (ROSC) after recent respiratory symptoms. The patient was subsequently found to have a subarachnoid hemorrhage and also tested positive for COVID-19.

## Case presentation

A 66-year-old female with a past medical history of diabetes, hypertension, and hyperlipidemia was brought to the emergency department (ED) post-cardiac arrest with ROSC. The patient had a one-week history of dry cough, shortness of breath, and general malaise. She had just returned from a month-long stay in the Dominican Republic five days prior to the onset of symptoms. She had not had any sick contacts within her travel group or where she had been staying while out of the country. On the day of the presentation to the ED, the patient’s son had found her on the floor next to her bed sitting upright. She had asked him to take her to the ED because of difficulty in breathing and he had called an ambulance. Before the paramedics had arrived, the patient had become unresponsive and stopped breathing. Her son had immediately started cardiopulmonary resuscitation (CPR). Paramedics had arrived on the scene about five minutes after the onset of bystander CPR and had found the patient to be in asystole. They had achieved ROSC after two administrations of epinephrine and seven additional minutes of CPR. The paramedics had informed us of COVID-19 risk factors prior to the arrival, and we wore appropriate personal protective equipment during patient encounters.

**Figure 1:**
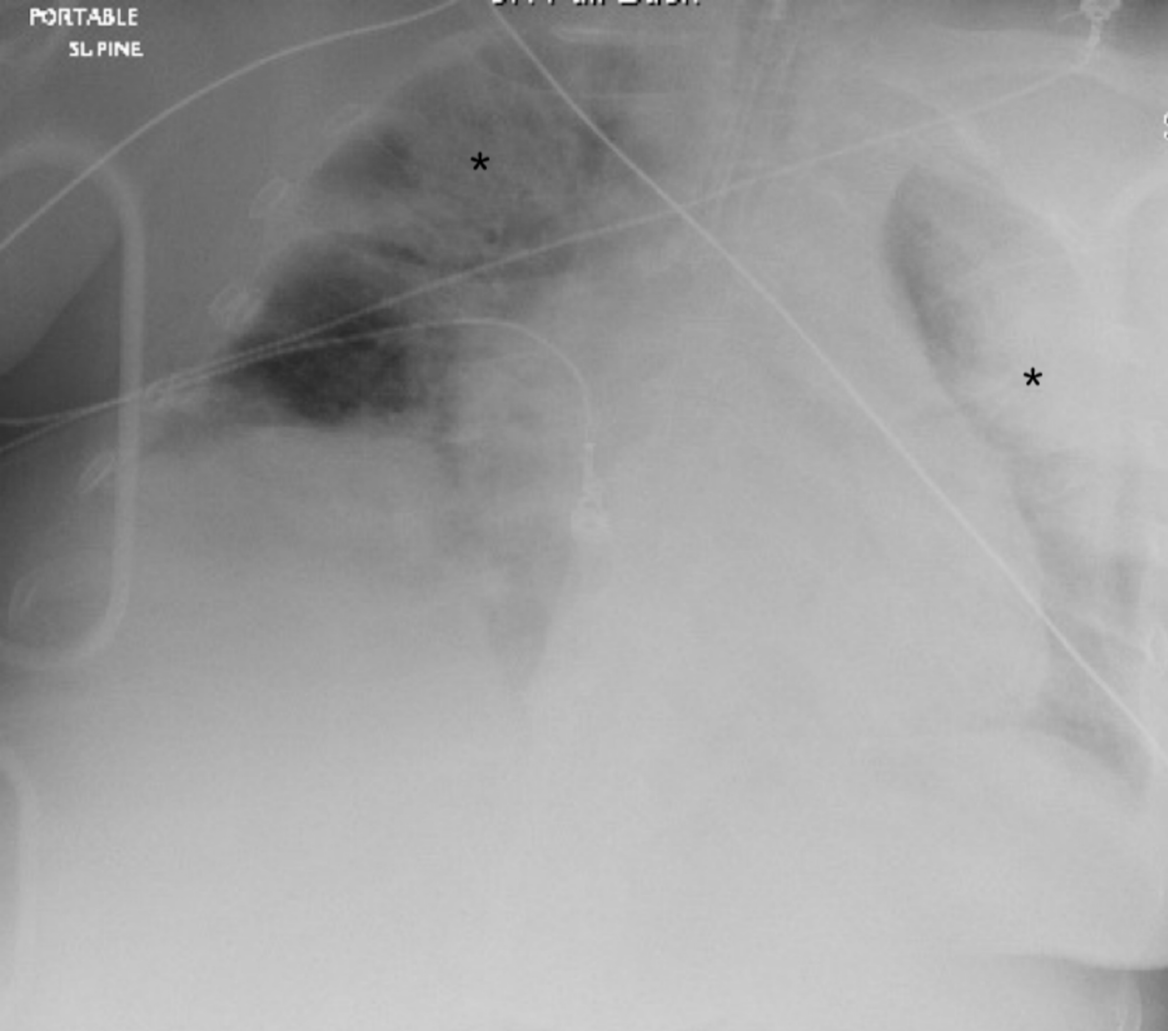
Chest X-ray showing bilateral infiltrates (asterisks)

On arrival to the ED, the patient was ventilated through a supraglottic airway with a right tibial intraosseous line in place. The patient again had a cardiac arrest on arrival with a pulseless electrical activity (PEA) rhythm noted on the monitor. She had multiple arrests during the initial presentation and an additional 11 minutes of CPR was administered. She received epinephrine, sodium bicarbonate, and calcium chloride during these resuscitations. The patient was intubated using video laryngoscopy and an emergent femoral central line was placed. She was started on a norepinephrine drip after the final ROSC in the ED. The patient was placed on a ventilator on volume control with positive end-expiratory pressure (PEEP) of 12 cmH2O. Arterial blood gas after intubation showed a pH of 6.78, pCO2 of 77.5, and a pO2 of 97 on 100% FiO2. A sodium bicarbonate drip was initiated. A chest X-ray showed bilateral infiltrates (Figure 1).

The patient was started on a sepsis protocol with intravenous vancomycin and cefepime. Given the preceding respiratory symptoms and imaging, a swab for SARS-CoV-2 was sent. The patient was not started on sedation in the ED as she remained unresponsive on examination. Initial labs showed elevated creatinine, troponin, and liver enzymes consistent with her cardiac arrest and shock. Her international normalized ratio (INR) was 1.2 and her platelets were within normal limits. Her white blood cells were elevated and her lymphocytes were within normal limits. Her lactic acid was 20.5 mmol/L. Her influenza testing was negative.

**Figure 2:**
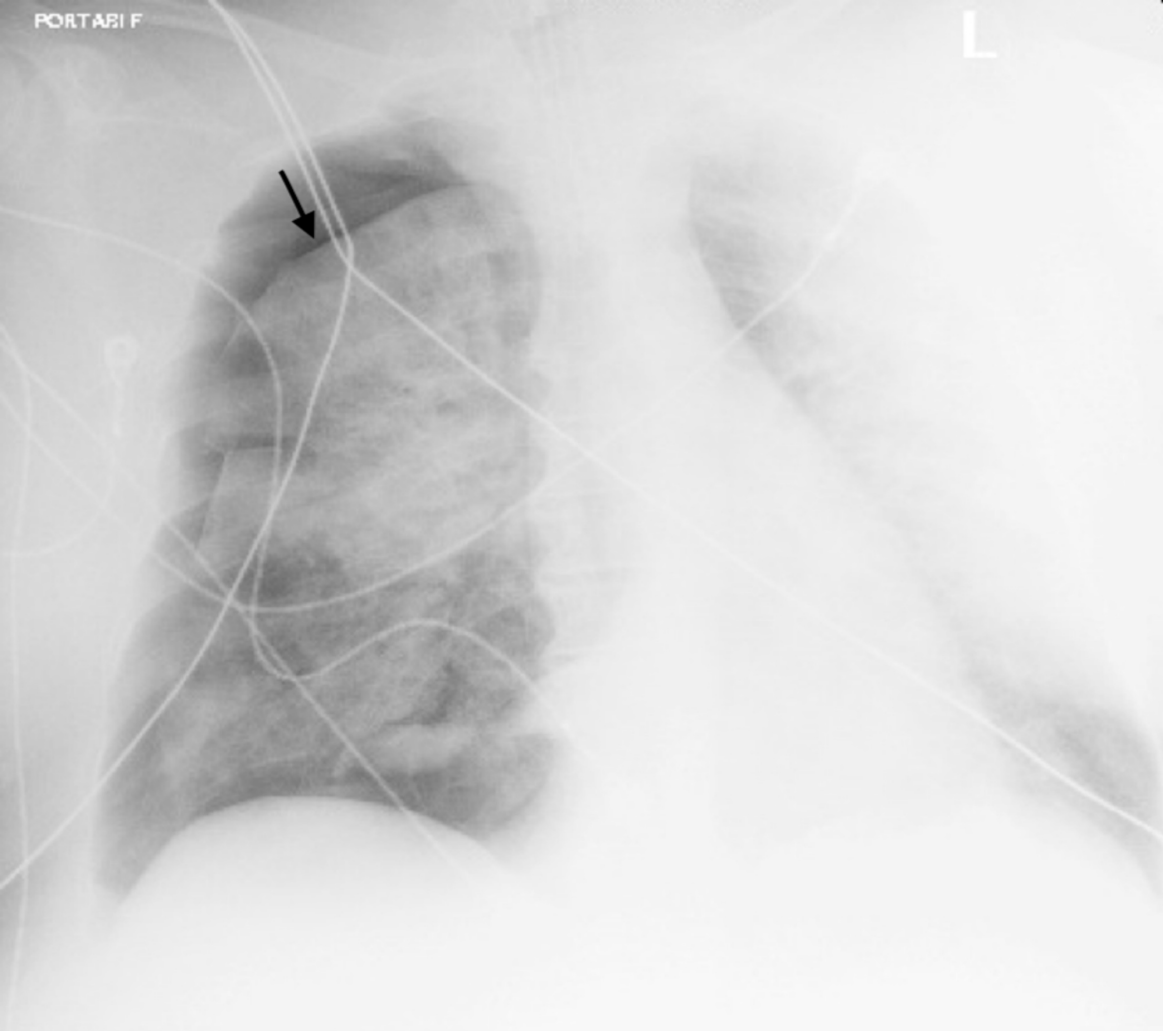
Chest X-ray demonstrating 30% pneumothorax on the right (arrow)

The intensive care team was consulted, but the patient had to remain in the ED due to a lack of bed availability in the intensive care unit (ICU). Approximately five hours after the initial intubation, the patient’s oxygen saturation dropped to around 80% on the ventilator and a chest X-ray revealed a 30% right-sided pneumothorax (Figure 2).

**Figure 3:**
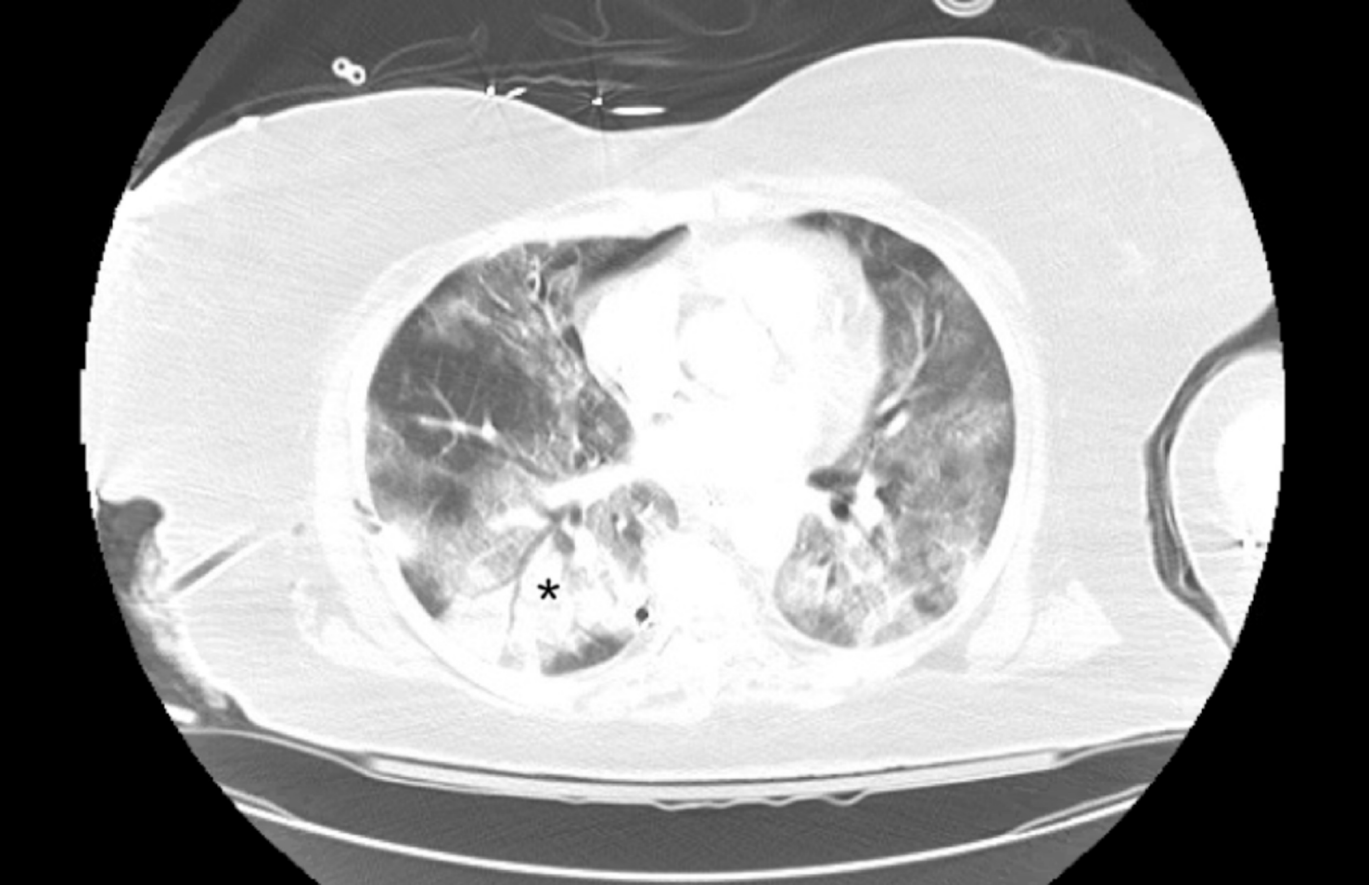
Chest CT demonstrating bilateral ground-glass opacities (asterisk), a pulmonary hallmark of COVID-19 infection CT: computed tomography

A 24-French chest tube was placed with the resolution of the pneumothorax. CT of the chest revealed a resolving right anterior pneumothorax with extensive bilateral ground-glass opacifications, considered a pulmonary hallmark for COVID-19 infection (Figure 3) [6].

**Figure 4:**
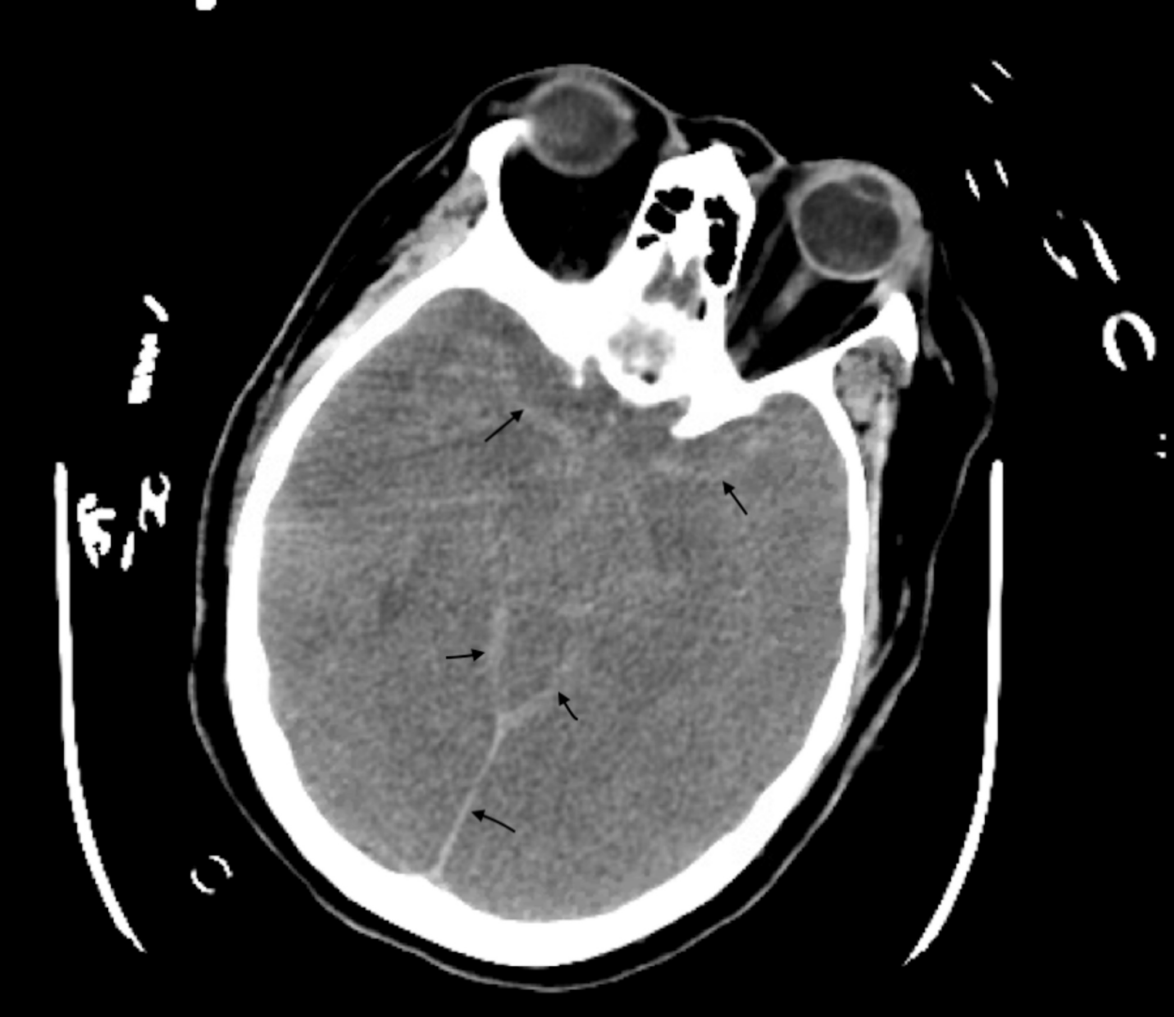
Brain CT demonstrating extensive subarachnoid hemorrhage (arrows) CT: computed tomography

CT imaging of the brain revealed extensive subarachnoid hemorrhage extending into the suprasellar cistern, Sylvian and interhemispheric fissures, effacement of the fourth ventricle, and diffuse cerebral edema (Figure 4).

A CT of the cervical spine was negative for fracture. Neurosurgery was consulted but no emergent surgical interventions were recommended given the poor neurological exam. She was given mannitol by the intensive care team and then started on hypertonic saline. They also started azithromycin and hydroxychloroquine for suspected COVID-19.

In the ICU, the patient was unresponsive to verbal or painful stimuli, had 6-mm fixed pupils nonreactive to light, absent corneal and gag reflexes, and absent oculocephalic and vestibulocephalic movements. A nuclear medicine brain flow scan showed the absence of intracranial cerebral perfusion, which supported the clinical diagnosis of brain death. The patient again had a cardiac arrest the next night and was pronounced dead. Her COVID-19 testing later returned positive.

## Discussion

The diagnosis and management of COVID-19 patients continue to evolve. Understanding the potential manifestations of the disease is an important aspect of early treatment. A study from Wuhan, China reported neurological symptoms in 36.4% of patients. These were described as acute cerebrovascular accidents including intracranial hemorrhage, impaired consciousness, and muscular injuries. These symptoms were more often noted in patients with severe disease. One out of the 214 patients included in the study were noted to have an intracranial hemorrhage [7].

**Figure 5:**
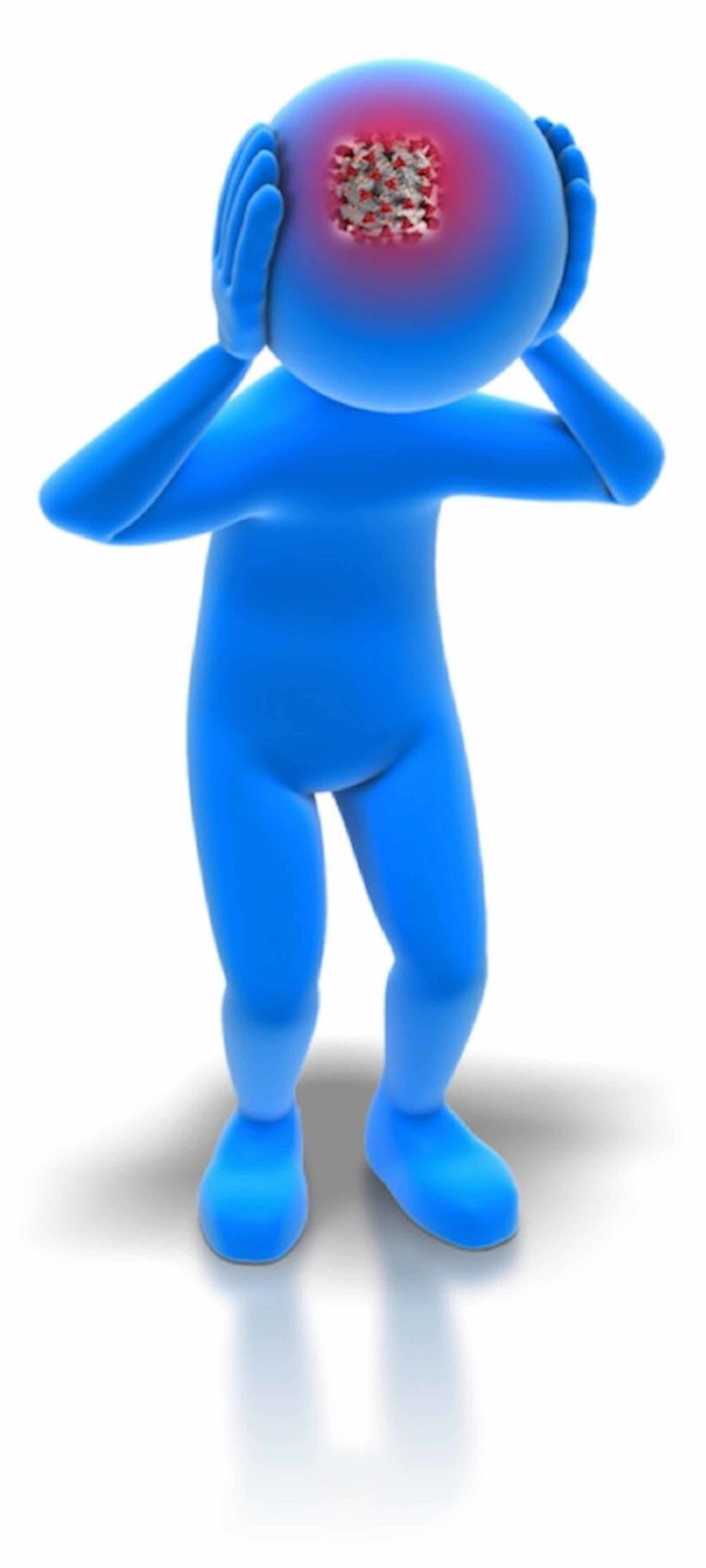
Artistic rendering of COVID-19 attacking the brain COVID-19: coronavirus disease 2019

There is a case report of an adult female with respiratory symptoms who developed altered mental status and was found to have acute hemorrhagic necrotizing encephalitis and positive SARS-CoV-2 testing [8]. They attributed this to a possible intracranial cytokine storm, which has been reported in COVID-19 patients with severe illness (Figure 5) [9].

Studies have also noted thrombocytopenia in these patients [5]. A patient in Thailand presented with petechiae only and later tested positive for COVID-19 [10]. A case report prior to this pandemic had linked immune thrombocytopenic purpura (ITP) to coronavirus [11]. However, our patient did not have thrombocytopenia on initial lab tests.

Intracranial hemorrhages appear to be a rare finding associated with COVID-19, with an unknown etiology. Our patient was initially found sitting next to her bed, although there was no reported head trauma or evidence of external trauma on examination. A pseudo-subarachnoid hemorrhage secondary to severe cerebral edema is also possible, although less likely, as evidence of subarachnoid hemorrhage was present on several CT slices [12]. Our patient had a one-week history of respiratory symptoms prior to her cardiac arrest and a subsequent diagnosis of subarachnoid hemorrhage. It seems that severe COVID-19 disease could be linked to intracranial hemorrhaging from cytokine storms or coagulation abnormalities. Regardless, it is important to consider severe neurologic disease in COVID-19-positive patients with altered mental status.

## Conclusions

Initially, the presentation of COVID-19 infection was thought to consist of fever, cough, and predominantly respiratory symptoms. However, as this case illustrates, severe neurological manifestations including intracranial hemorrhages should be considered in COVID-19-positive patients with altered mental status.
